# Developing and Testing a Reflection Method for Implementation of a Professional Reporting Guideline in Community Nursing: Design‐Based Research

**DOI:** 10.1111/jan.16747

**Published:** 2025-01-21

**Authors:** Nicole Vullings, Maud Heinen, Marian Adriaansen, Hester Vermeulen, Philip Van Der Wees, Marjo Maas

**Affiliations:** ^1^ Radboud Institute of Health Sciences, Scientific Institute for Quality of Healthcare Radboud University Medical Centre Nijmegen The Netherlands; ^2^ Faculty of Nursing Studies HAN University of Applied Sciences Nijmegen The Netherlands

**Keywords:** clinical Guidelines, community Care, nursing Home Care, reflective Practice, research Implementation

## Abstract

**Aim:**

To develop a reflection method for community nurses and certified nursing assistants to support the implementation of a professional reporting guideline for nurses and certified nursing assistants in daily care and to identify its key features.

**Design:**

Design‐based research.

**Methods:**

This study was conducted in the Netherlands from February 2021 to April 2022. The reflection method was developed by a design group (community nurses, certified nursing assistants and a patient representative) and four test groups of nurses. Experiences of participants were explored with video recordings and observational notes from test group meetings. The data were thematically analysed to refine the reflection method and identify key features.

**Results:**

A final reflection method was developed. We identified three main themes: (1) Impact on behaviour change, (2) group learning and (3) conditional factors for critical reflection. Seven key features emerged as essential, forming the building blocks of the reflection method: focus on critical reflection, allocate time to formulate themes, include participants from various backgrounds and organisations, ensure the group is appropriately sized, allow for sufficient time, keep it simple and attractive and stimulate the group to make the transfer of learning to their clinical practice.

**Conclusions:**

The final method included two 2 h meetings with up to six participants and a coach. Participants learned from critical reflection and feedback to improve the reporting quality and critical assessment of daily practices, especially from colleagues with varying team affiliations and educational backgrounds.

**Impact:**

This reflection method enables community nurses and certified nursing assistants to learn collaboratively, aligning with the ‘Nursing and Caring Reporting’ guideline and bridging the gap between research and clinical practice.

**Report Method:**

The COREQ guideline was used.

**Patient or Public Contribution:**

The study design facilitated close collaboration among researchers, community nurses, certified nursing assistants and clients.

**Summary**

- A reflection method for registered community nurses and certified nursing assistants to enhance reporting in line with guideline recommendations.
- Key features of a reflection method, such as stimulating participants to make the transfer of learning to their own clinical practice.
- Reflection on the use of a professional reporting guideline encourages community nurses and certified nursing assistants also initiate the evaluation of care provided.

## 1 Introduction

Clinical practice guidelines have been developed to support healthcare professionals in making clinical decisions, reduce unwanted practice variation and promote patient well‐being, thereby optimising the quality of client care (Graham et al. 2011; Spoon et al. 2020; Wensing, Grol, and Grimshaw 2020). In recent years, clinical guideline development for nurses in Europe has accelerated (Spoon et al. 2020). These guidelines are intended for nurse practitioners, registered nurses and certified nursing assistants. However, they experience barriers to adopting guideline recommendations in clinical practice (Arts et al. 2016; Fischer et al. 2016). Clinical guidelines are designed to support decision‐making, but they are not tailored to the specific target groups for which they provide care (Flottorp et al. 2013; Magwood et al. 2020; Ovretveit et al. 2018; Pfadenhauer et al. 2017). Time pressure necessitates a quick assessment of situations and fast decision‐making. Adequate decision‐making demands flexible knowledge, appropriate reasoning strategies and self‐confidence (Fischer et al. 2016; Higgs, Loftus, and Christensen 2018). In addition to the time pressure issue, clinical guidelines are often perceived as too extensive, hindering the clinical reasoning and decision‐making process (Arts et al. 2016; Correa et al. 2020; Fischer et al. 2016).

To improve the adequate use of guidelines, strategies must be employed to overcome the barriers to guideline implementation. A strategy to enhance the application of nursing guidelines in clinical practice is to critically reflect on and discuss the quality of client care based on the recommendations in guidelines (Wensing, Grol, and Grimshaw 2020). Critical reflection allows for the discussion of complex and unfamiliar clinical situations in healthcare (Rolfe 2014). Research indicates that reflective practice enhances behaviour change in clinical guideline adherence and improves nurses' self‐understanding by attributing meaning to their experiences (Goulet, Larue, and Alderson 2016). Additionally, it is highly esteemed by professionals, fostering long‐term changes in clinical practice through increased learning, competency and self‐awareness (Asselin and Fain 2013; Choperena et al. 2019; Contreras et al. 2020; Patel and Metersky 2022).

Previous research on reflective practice has been conducted among primary healthcare physical therapists to promote clinical reasoning and acting in accordance with practice guidelines (Maas et al. 2015). The studied reflection method proved to be feasible and effective. In this method, healthcare professionals evaluated the quality of their own work and that of their colleagues on the basis of predetermined criteria and gave each other constructive feedback. Experiences with Maas's method seem to be a good basis for developing, testing and evaluating a reflection method to improve the adherence of nurses to guidelines.

## 2 Background

Adequate nurse reporting is a crucial basis for the nursing process, ultimately leading to improved quality of care for clients (De Groot et al. 2019). Nurse reporting and documentation in a client report are important indicators of effective client care delivery (De Groot et al. 2019). High‐quality reporting is important for the assessment, provision, evaluation and transfer of care, with the aim of promoting continuity and quality of care (Akhu‐Zaheya, Al‐Maaitah, and Bany Hani 2018).

To improve the quality of nurse reporting, a professional reporting guideline for nurses and certified nursing assistants was developed. This guideline was developed by the Dutch professional nursing association (V&VN 2022). This organisation focuses on supporting and empowering its members by advocating for their interests, promoting professional standards and developing guidelines and quality norms and also provides a platform for knowledge sharing, networking and professional development.

The aim of the Reporting guideline for nurses and certified nursing assistants is to standardise nursing reporting practices to improve the quality, continuity and safety of patient care. This guideline outlines essential reporting standards and provides a framework for consistent and accurate record‐keeping. Nurses play a critical role in implementing these standards because high‐quality reporting supports effective communication, informed decision‐making and enhanced patient outcomes (V&VN 2022).

However, after the development of the Reporting guideline for nurses and certified nursing assistants, implementing the recommendations of this guideline in practice proved challenging.

In community nursing in the Netherlands, registered nurses and certified nursing assistants collaborate within self‐directed teams (De Groot et al. 2022). Certified nursing assistants carry out caregiving tasks according to a care plan designed by a community nurse, but both roles involve making autonomous decisions in daily care situations. Like many healthcare professionals, they encounter challenges in implementing guideline recommendations (Fischer et al. 2016; Higgs, Loftus, and Christensen 2018). Therefore, a structured reflection method is crucial to support them in effectively utilising guideline recommendations in routine practice. This approach aligns with evidence suggesting that reflective practices can enhance clinical reasoning, support decision‐making and foster adherence to clinical guidelines, ultimately improving the quality of care (Contreras et al. 2020; Wensing, Grol, and Grimshaw 2020).

## 3 The Study

### 3.1 Study Aim

To develop a reflection method for community nurses and certified nursing assistants to support the implementation of a professional reporting guideline for nurses and certified nursing assistants in daily care and to identify the key features.

The aim of this study was two‐part: the first was to develop a reflection method through a design‐based research, and the second was to achieve a theoretical outcome by identifying its key features (Armstrong, Dopp, and Welsh 2022) in order to make it possible to transfer the reflection method to alternative guidelines or contexts.

### 3.2 Study Design

A design‐based research approach was employed to develop the reflection method and to identify its key features (Reeves and McKenney 2015). Key features were defined as the building blocks of the reflection method. By identifying these key features, it became clear what worked and what did not when using the reflection method in practice (Greenhalgh et al. 2023; Wensing, Grol, and Grimshaw 2020). This design was chosen to optimally align with the different needs and abilities of certified nursing assistants and community nurses as they implemented guideline recommendations in daily practice. Design‐based research has increasingly gained popularity in healthcare and welfare due to the complexity of care issues in the healthcare sector, as conducting research in the context and iteratively designing methods yielded authentic and actionable knowledge (Armstrong, Dopp, and Welsh 2022; Przybilla et al. 2018). Action research and design‐based research are similar in many aspects, but design‐based research was specifically chosen for this study because the primary goal was to develop a new method rather than to induce change in clinical practice (van Lieshout, Jacobs, and Cardiff 2021). Design‐based research focused on developing an effective method or intervention and discovering new principles on what did and did not work by employing multiple iterations of development, testing, evaluation and implementation in close collaboration with key stakeholders (Peffer and Renken 2016). Because of its flexible, iterative and co‐creative approach, design‐based research was deemed a suitable approach to develop a reflection method that worked for both nurses and certified nursing assistants (Peffer and Renken 2016; Van t Veer et al. 2020).

### 3.3 Study Setting

This study was conducted in the Netherlands and involved a 15‐month process with two cycles of prototype development from February 2021 and April 2022.

### 3.4 Participants and Recruitment

Participants included community nurses, certified nursing assistants from home care organisations in the Netherlands and one patient representative. They were invited to participate in either a design group or a test group. Forty randomly selected home care organisations across the Netherlands were eventually invited to participate in this study, of which four agreed to take part. The participating home care organisations were located in different regions across the Netherlands.

From each of the four participating organisations, a convenience sample of community nurses and certified nursing assistants was obtained through invitations sent via the organisations' newsletters and email. Those who responded received an information letter and an informed consent form from the first author (NV). After receiving signed informed consents, the researcher assigned participants to the design and test groups, ensuring an equal distribution of community nurses and certified nursing assistants. Three groups worked closely together in this study: a design group (*n* = 9), consisting of community nurses, certified nursing assistants and a patient representative; four test groups, each including six participants (community nurses and certified nursing assistants); and the research group (NV, MM, MH, MA, HK and PW).

The design group included one community nurse and one certified nursing assistant from each home care organisation, while each test group consisted of three community nurses, three certified nursing assistants and a coach to facilitate the reflection process during test meetings. The coach was a community nurse from the participating organisation, trained for this role. The patient representative was approached through an independent consultancy office that assists organisations in promoting equitable cooperation between patients and healthcare professionals at an organisational level (IKONE n.d.).

The research group consisted of experts in the field of guideline development, community care, nursing research, educational research and reflective practice The research group monitored the development process. Members of the research group (NV, MM and MH) guided the design and test groups closely.

### 3.5 Study Procedures

#### 3.5.1 The Cyclical Process

**FIGURE 1:**
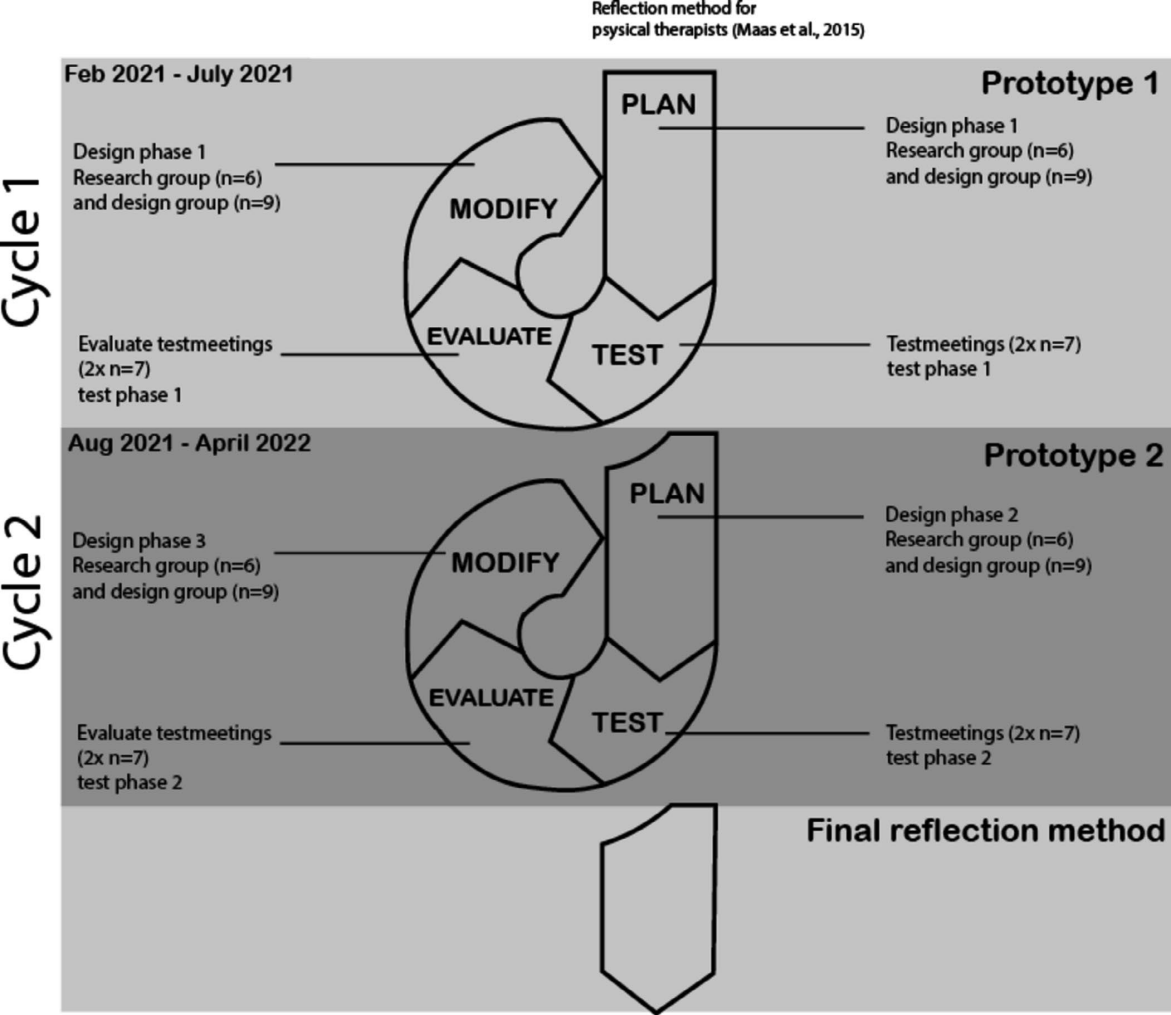
Schematic view of the study: 15‐month process of two cycles.

This cyclical process aligned with the iterative model proposed by McKenney and Reeves (2012), which incorporated three core processes: (1) analysis and exploration, (2) design and construction and (3) evaluation and reflection (McKenney and Reeves 2012). These three processes were ensured in the cyclical process of this study through the iterative use of design and test phases. In the design phases, the focus was on exploring and developing prototypes of the reflection method with participants (co‐designers). In the test phases, the focus was on testing, evaluating, reflecting and modifying the method design. This 15‐month cyclical process comprised three design phases and two test phases (see Figure 1), with each phase lasting for 3 months.

This study was conducted across two test phases and three design phases. The test and design meetings took place during participants' working hours. Table 1 presents an overview of the participants, aims and intended outcomes for phases one and two.

**TABLE 1:** Overview of the aims and number of meetings, participants and intended outcomes for each phase.

Phase	Number of meetings	Aim	Participants	Intended outcome
Design phase 1 (prototype)	*n* = 1	To probe the first meeting with the design group	Research group (*n* = 6)	Appropriate design methods and materials
	*n* = 2	To develop quality criteria for nurse reporting	Design group and research group: 1. Community nurses (*n* = 4), certified nursing assistants (*n* = 4), patient representative (*n* = 1). 2. Research group representatives (*n* = 3)	Criteria for nurse reporting
	*n* = 1	To identify key features of a good reflection method and develop the first prototype	Design group Community nurses (*n* = 4), certified nursing assistants (*n* = 4), patient representative (*n* = 1)	Prototype 1 of the reflection method Manual for prototype 1
Test phase 1	*n* = 2	To test and evaluate the first prototype and identify key features	Test group and Research group 1. Community nurses and certified nursing assistants, including one designated coach (*n* = 7). 2. Representatives of the research group (*n* = 3)	Strengths and weaknesses of the first prototype
Design phase 2 (refine)	*n* = 1	To make an overview of the important yields of the test meetings To probe work formats for the design group	Research group 1. Representatives of the research group (*n* = 3) 2. Research group (*n* = 6)	An overview of important yields Appropriate design methods and materials
	*n* = 1	To create more depth to the reflection method so the focus is on what the professional needs to learn	Design group (*n* = 9) Community nurses (*n* = 4), certified nursing assistants (*n* = 4), patient representative (*n* = 1)	Prototype 2 of the reflection method Manual for prototype 2
Test phase 2	*n* = 2	To test and evaluate the second prototype and identify key features	Test group and Research group 1. Test group (*n* = 7) Community nurses and certified nursing assistants including one designated coach (*n* = 7). 2.Representatives of the research group (*n* = 3)	Strengths and weaknesses of the second prototype
Design phase 3 (modify)	*n* = 1	To present the final design and to modify where necessary	Design group and Research group 1. Design group (*n* = 9) 2. Representatives of the research group (*n* = 3)	Final reflection method Manual for the final reflection method

Seven of the eight test group meetings took place online due to COVID‐19 restrictions, as did all five design meetings.

#### 3.5.2 Design Phase 1 (Prototype 1)

The existing reflection practice for physical therapists was used as a basis for the design (Maas et al. 2015; van Dulmen et al. 2014). In this reflection method, physical therapists evaluated the quality of their own work and that of their colleagues on the basis of predetermined quality criteria and provided each other feedback in an online learning environment. They reflected on the results in in‐person meetings facilitated by a coach.

In the first design phase, the criteria for reporting according to a Reporting guideline for nurses and certified nursing assistants were developed in two meetings. Table 2 presents the nurse reporting quality criteria. The quality criteria were perceived as a simple and quick way to critically assess the quality of client reports and to identify opportunities for improvement. In order to ensure that all participants understood the criteria, each criterion was given a brief explanation and example.

**TABLE 2:** Final criteria for good reporting for registered nurses and certified nursing assistants.

Content criteria
1.1. The client file contains all relevant information about the client, including their social context, current health and personal history
1.2. Objectives are formulated in a specific, measurable, acceptable, realistic and time‐dependent manner
1.3. All phases of the nursing process are reported in the client file
1.4. Current or potential health problems are identified, and subsequent actions, agreements and evaluations are described and adjusted as necessary
1.5. The development of the client's health is monitored, evaluated where necessary by means of valid measuring instruments and clearly presented
1.6. The report shows that objectives and evaluations have been discussed with the client or their relatives and that they have been involved in any decision‐making
Form criteria
2.1. The client file is written in understandable language and is accessible to the client and/or their relatives
2.2. The reporting is succinct and contains no superfluous or repeated information
2.3. Subjective findings and objective observations can be clearly distinguished from each other

In a third meeting, the *cultural probe method* and *mind mapping* were used to identify important elements of an effective reflection method (Burrows, Mitchell, and Nicolle 2015). This method stimulates creativity and offers people insights into the preferences and desires of professionals and clients regarding adequate reporting and good reflection practice (Burrows, Mitchell, and Nicolle 2015; Wherton et al. 2015). Design phase 1 resulted in the first prototype of the reflection method.

At the end of design phase 1, the researchers (MH, MM and NV) prepared a manual for the participants and the coaches describing the reflection method. The manual covered the preparation, the content of the meetings, a time schedule and the actions taken after the meetings. The coach's manual additionally contained practical tips for effective coaching.

#### 3.5.3 Test Phase 1

The coaches were trained at the start of the test phase by the research team (MM, NV). The initial training lasted 1.5 h. The first part of the training focused on the core principles of effective communication and constructive feedback. Furthermore, dedicated attention was directed towards the sequential stages of the reflection method that had been developed. The remaining time was used to practice these steps through a case.

After the training, the coaches were used during test meetings. In this first test phase, two home care organisations tested prototype 1. Two hours were scheduled for each test meeting with six participants and a coach. In one test group, six participants from one team participated, and in the other test group, six participants from different teams within one organisation participated.

After each test meeting, there was a post‐discussion moment with one researcher (NV) and the coaches to discuss experiences and any potential difficulties.

#### 3.5.4 Design Phase 2 (Prototype 2)

The second design phase consisted of a 2 h meeting in which the design group with the researchers (NV, MH and MM) identified how the method could be improved. The main question addressed was “How can more depth be achieved in the reflection method so that the reflection is more directed at what the professional needs to learn?” Therefore, during design phase 2, in‐depth questions and open‐ended questions were deliberately asked (Bellersen and Kohlmann 2016; Niezink et al. 2017). The ‘five times why’ methodology was used for identifying the true, deeper cause of a problem (Niezink et al. 2017). To provide more space to questions that relate to both the nurse and the context of the case, a combination of ‘why’ questions and other open‐ended questions was chosen.

#### 3.5.5 Test Phase 2

In the second test phase, the other two organisations tested prototype 2. Two hours were scheduled for each test meeting with eight participants and a coach. Two additional participants were added to the groups because six participants appeared vulnerable, as well as to mitigate risk due to illness, priorities in work or holidays.

In test phase 1, participants indicated that joining with others from different teams was valuable. Therefore, in test phase 2, the test groups contained participants from the various teams.

#### 3.5.6 Design Phase 3 (Final Reflection Method)

Three researchers (NV, MH and MM) from the research group reviewed the proceedings of all design and test meetings and suggested modifications to prototype 2. During the third design meeting with the design and research group, the modifications were presented to all participants to reach consensus on the final prototype.

### 3.6 Data Collection

Different data collection methods were used: video recordings of design‐ and test meetings including the evaluations and observation notes. All meetings of the design‐ and test groups were video‐recorded, and informed consent was obtained from each participant. Following the meetings, the recordings were stored in a secured locked Cloud at the HAN University of Applied Sciences (Nijmegen) that only the researchers (NV, MH and MM) had access to. The video recordings of the design meetings lasted between 100 and 130 min and the test meetings between 100 and 120 min.

At least two researchers (MH, MM and NV) were present at the design and test meetings and made notes on their individual observations of the participant behaviour. At the end of each test meeting, the researchers evaluated the meetings with participants to identify the strengths and weaknesses of the tested prototype. This evaluation was also video‐recorded, and notes were made.

An overview of the important yields of the test meetings was produced by the researchers (NV and MH). The participants of the test groups received this overview by mail, asking if the data had been accurately interpreted from the test meetings and whether any items were missing or needed to be added, as part of the member‐checking process (Morse 2015).

### 3.7 Data Analysis

All data from the design and test meetings were iteratively collected and analysed so that relevant information for the improvement of the design could be identified and used to modify the reflection method for the next design phase.

#### 3.7.1 Analysis of the Notes of Design and Test Meetings

The principal investigator (NV) compared the notes of MH and MM, and differences relevant to the design of the reflection method were discussed. Consensus between the three researchers was reached. When the notes were regarded useful for the design, these parts of the video recordings of the meetings were studied again and discussed to better understand the behaviour of the participants and the group dynamics.

#### 3.7.2 Analysis of the Video Recordings

An external company, who signed a non‐disclosure agreement, transcribed non‐verbatim the video recordings. The test and design meetings were conducted and analysed in Dutch. The quotes in the results were translated into English by a researcher and verified by an independent translation agency.

The transcripts were analysed using thematic analysis, as suggested by Braun and Clarke (2021). The first transcript was studied and coded independently by the first author (NV) and two other researchers (MH and MM), in Atlas TI 22. Initial codes were applied to the data that were deemed relevant for the reflection method design. By comparing codes, a code‐book was developed and new codes were added or modified where necessary. Consequently, all data were analysed. The researchers compared and discussed the codes and finally agreed on sub‐themes and themes. After that, the researchers iteratively discussed the data from the design‐ and test meetings, the sub‐themes and themes. They critically reflected on their perspectives and preconceptions as researchers and finally on a set of key features of the reflection method that emerged from the data. Through these key features, it has been clarified which elements are important to work with a reflection method to ultimately act more according to the guideline.

Reflexivity and independent coding enhanced the trustworthiness of the study findings (Busetto, Wick, and Gumbinger 2020).

### 3.8 Ethical Considerations

This study adhered to the ethical principles of the Declaration of Helsinki. Ethical approval for this study was granted by the local ethical research committee of the HAN University of Applied Sciences Nijmegen (Ethical Research Committee, ref.no. ECO 235.02/21). Participation in this research was voluntary, and the researchers maintained the participants' right to withdraw from the research.

### 3.9 Rigour and Reflexivity

The study report used the Consolidated Criteria for Reporting Qualitative Research (COREQ). Credibility was enhanced through methodological triangulation, utilising a combination of data resources. Additionally, the reflection method had been developed from different perspectives, with critical reflection applied to each of these perspectives (Korstjens and Moser 2018). The research team consisted of members with diverse expertise (guideline development, community care, nursing research and reflective practice), and the participating registered nurses and certified nursing assistants had varied backgrounds in areas such as work experience and educational level. In addition, data analysis and its interpretation was conducted with multiple researchers (NV, MM and MH) to ensure credibility (Busetto, Wick, and Gumbinger 2020).

## 4 Results

### 4.1 Sociodemographic Characteristics

Nurses and certified nursing assistants (*n* = 33) participated in the study. Table 3 presents the background characteristics from the nurses and certified nursing assistants.

**TABLE 3:** Participant characteristics of the study population.

	*n* (%)
Total	33
Gender	
Male	3 (9%)
Female	30 (91%)
Working hours	
< 16 h	0 (0%)
16–24 h	27 (82%)
25–32 h	4 (12%)
> 32 h	2 (6%)
Working experience	
0–3 years	6 (18.2%)
4–6 years	6 (18.2%)
7–10 years	3 (9.1%)
11–20 years	8 (24.2%)
> 20 years	10 (30.3%)
Educational level	
Community Registered Nurse	12 (36.4%)
Registered Nurse	8 (24.2%)
Certified Nursing Assistant	13 (39.4%)

### 4.2 Themes

Three main themes including eight sub‐themes were identified. Table 4 presents the sub‐themes, themes and key features. The sub‐themes and themes are described below. Quotes were added to support and illustrate the findings within each sub‐theme.

**TABLE 4:** Main themes, sub‐themes and key features of the data.

Themes	Sub‐themes	Key features
1. Impact of the reflection method on behaviour change	Awareness of quality standards for reporting	1. Help participants to focus on critical reflection on their own reporting behaviour instead of talking about the context of the client^a^
	Behaviour change regarding reporting	2. Allocate time to formulate an overarching theme or problem that aligns with the learning needs of the entire group
	Critical view on nursing practice	1. Help participants to focus on critical reflection on their own reporting behaviour instead of talking about the context of the client^a^
2. Impact of the reflection method on group learning	Team spirit and communication	3. Include participants from different teams, varying in working experience and educational levels 4. Limit group size to six participants
	Professional development	3. Include participants from different teams, varying in working experience and educational levels
3. Conditional factors for critical reflection	Simple and plain language	5. Keep the reflection method simple and attractive for all participants
	Time for reflection	6. Allow for sufficient time for clarification of learning questions and reflection time
	Coach support	7. Stimulate the group to make the transfer of learning to their own clinical practice

^a^ Key feature 1 is connected to two sub‐themes.

#### 4.2.1 Impact of the Reflection Method on Behaviour Change

The reflection method impacted on several levels of change: Awareness of quality standards for reporting, behaviour change regarding reporting and a critical view on nursing practice.

##### 4.2.1.1 Awareness of Quality Standards for Reporting

Using the quality criteria for good reporting when assessing own client reports and those of colleagues was perceived as a helpful tool to enhance reflection and to provide feedback.

Participants indicated that the reflection method caused them to be more aware of their reporting routines in clinical practice and stimulated them to look more critical at a client report.

R6Q1: Sometimes you work on autopilot, especially when it's busy and clients have been in care for a long time. At times, you may think everything is fine. However, by critically appraising [nursing reports] with others, you realize that it's not always right. That is an eye‐opener.

R4Q1: As I reviewed the criteria and we discussed my client's file together, it suddenly became clear. That client should have been out of care a long time ago. While it doesn't directly relate to the client file directly, it was very important for me.

##### 4.2.1.2 Behaviour Change Regarding Reporting

Behaviour change regarding reporting refers to how the reflection method alters participants' reporting habits in clinical practice. The observations of the test meetings pointed out that participants sometimes spent too much time on talking about details of the clients’ condition and their social contexts instead of reflecting on the quality of the clients’ record.

Res1Q1: The discussions among the nurses were so focused on the client's context that the criteria for proper reporting and the guidelines itself were either overlooked or only addressed occasionally.

In addition, by focusing on details of the clients’ condition and their social contexts, the discussion lost its relevance for the entire group as participants failed to identify learning objectives for their own reporting competencies.

R1Q1: Context: A participant shares detailed information about the client. Another participant responds: ‘Maybe it is an idea to have a conversation with the client. Well, I do not know the client and the client situation which makes it hard to talk about it’.

To help the group in focusing on the quality of the client record and involving the entire group in the discussion, the second prototype of the reflection method was modified. After the presentation of the client record, time was allocated to formulate an overarching theme or problem on a more conceptual level that applied to all participants. This overarching theme aligned with the learning needs of the entire group. In test phase 2, this modification proved to be valuable.

In addition, participants indicated that the discussion about the overarching theme or problem also resulted in concrete actions to improve the client reports and change their daily reporting routines.

R32Q1: Because the group in the reflection method consisted of colleagues inside and outside the team, discussions sometimes included different perspectives and practices. This did make me think about my own methods and whether things could be done differently.

Using the reflection method to receive critical feedback from a colleague helped participants address the difficulties they experienced in reporting in clinical practice. The participants also reported that receiving feedback made them more critical of themselves and their colleagues.

R2Q1: Looking critically at yourself, but also at others. Back off the autopilot.

R18Q1: Now I read care plans more often than before. I also take a look at someone else's client report more often to see how she reports.

R19Q1: Through the feedback I received from my colleague, I found out that there were goals in the client report that were no longer current. Therefore, I started talking to the client again to review the care goals and schedule a new evaluation moment with the client.

##### 4.2.1.3 Critical View on Nursing Practice

The reflection method also provided a critical view of the overall nursing care. Participants noted that the feedback, along with the discussions that arose during the meetings, led to critical thinking and actions being implemented across all aspects of client care, not just in the reporting.

#### 4.2.2 Impact of the Reflection Method on Group Learning

The video recordings and observation data showed that the group and the coach impacted on several levels: Team spirit and communication and professional development.

##### 4.2.2.1 Team Spirit and Communication

Critical reflection with each other was considered even more important because of the isolated nature of the daily work of community nurses and nursing assistants. Meeting each other during the test meetings was perceived as having a positive impact on the team spirit.

R2Q2: I realized through these meetings how little we see of each other. It would be good if we could meet more often between care activities to catch up, but also to discuss more client cases.

The participants within a regular team as well as within mixed teams indicated during the evaluation that they felt sufficiently safe to express themselves and did not feel judged by colleagues during the face‐to‐face meetings.

The participants suggested that a group size of approximately six participants is optimal for fostering in‐depth reflection and ensuring enough time for discussion.

##### 4.2.2.2 Professional Development

The researchers' video recordings and observations of the test meetings showed that teams from the same organisation tended to focus on discussing current practices and specific client contexts, occasionally overlooking the core objective of reflecting on their own reporting behaviour. In contrast, mixed teams with nurses from different educational levels and different organisations were more successful in aligning with the reflection method's goals. Participants reported that reviewing client reports from teams of different organisations provided examples of different reporting styles, which inspired and motivated them to reflect on, adapt and enhance their own approaches.

R7Q1: It is nice to have a look behind the scenes of another team. Of course, this is also about looking more critically at yourself, a fresh perspective.

R17Q1: I think it's great that something is finally being developed that involves different levels. After all, you are jointly responsible for a client's care. By doing this together you put everyone more in their power.

#### 4.2.3 Conditional Factors for Critical Reflection

To be able to critically reflect on the quality of client reports, the data showed that participants had a number of needs: Use of simple and plain language, time for reflection, coach support, face‐to‐face contact and digital support.

##### 4.2.3.1 Simple and Plain Language

The video recordings and observations of the researchers showed that a number of certified nursing assistants struggled with formulating personal learning questions and personal action plans as part of the reflection method. These difficulties arose mainly because of unfamiliar terms such as ‘reflection’ and ‘learning questions’. In addition, they had difficulty understanding the criteria for good reporting.

R15Q1: To be honest, I understood very little of some of the criteria, for example, in the area of legislation and the nursing process. I asked some other certified nursing assistant colleagues, but several did not understand.

To help participants in understanding the criteria, the language has been clarified and simplified in test phase 2. Some of the participants suggested to simplify certain steps of the reflection method, word choices and suggested adding a short explanation and an example for each criterion.

In test phase 2, this modification proved to be valuable. The second set of quality criteria was perceived as a simple and quick way to critically assess the quality of the client file and to identify parts of the file that need further attention. Although the criteria and steps were simplified, a number of participants indicated that they needed support from the group. Simplifying the language combined with the support of the group seems to ensure that participants were able to follow the reflection method and put actions into practice.

R26Q1: To be perfectly honest, I was very nervous at the start. Everything was new. I was educated a very long time ago, and back then, there was no focus on reflecting and evaluating care plans within client reports. But because the new reflection method consists of fewer steps and the groups helped me, it worked. We really embraced it together.

##### 4.2.3.2 Time for Reflection

The video recordings and observation of the researchers showed that, because of the many steps in the first prototype, little time was left for discussion, and if so, the discussion was dominated by the same participants. As a result, the transfer to meaningful learning activities and reporting behaviour was limited.

Some participants reported that they needed time for reflection to adequately respond to questions posed during the meetings. They felt that some other participants answered immediately, hindering the process of sharing knowledge from their side.

R24Q1: I can't think as quickly as some others. They have already answered before I have had time to think it through.

To allow more time for discussing and refining their initial learning questions and for reviewing their action plans to facilitate the transition to meaningful learning activities and behavioural changes related to reporting, the second test phase was modified.

In the second prototype of the reflection method, specific time slots were scheduled to clarify and rephrase the learning question, to individually reflect on the learning question before responding and to discuss relevant learning goals and action plans. Furthermore, various steps have been merged or removed to make it more organised and clear for participants and to allow more time for discussion.

This modification was perceived valuable. Some participants indicated that they now felt they had more mental space for thought and input, allowing them to better link their learning experiences to their daily practice.

##### 4.2.3.3 Coach Support

The coaches who guided the group during the meetings indicated that they felt tense because of their lack of prior coaching experience and the novelty of the reflection method. In addition, they struggled to find a balance between keeping distance in their role as coaches and actively engaging in substantive contributions.

The observations of the researchers also revealed that in their attempt to adhere to the steps of the reflection method, the coaches sometimes did not pay sufficient attention to the group process resulting in insufficient group guidance. For example, discussions occasionally prolonged excessively, or insufficient support was provided in formulating effective goals. As a result, the aim of the meeting was occasionally missed.

Res2Q1: The coach finds it challenging to steer the process during the meeting when necessary. When someone picks up the phone during the digital meeting, it becomes chaotic, but the coach does not intervene. Similarly, when participants start discussing client details again, the coach does not always step in.

All coaches and participants agreed that involving an external coach from outside the organisation was not desirable. They preferred a coach that is familiar with the organisation's working methods, for example, the electronic health record system used.

### 4.3 Key Features

Seven key features of the reflection method emerged from the data. Table 4 presents the key features. One key feature is connected to two sub‐themes.

### 4.4 Final Design

The final design of the reflection method for guideline‐based reporting is displayed in Table 5. Each participant evaluates one client report of their own and gives feedback on two client reports of colleagues. Participants reflect on the feedback during the meeting and formulate one or more questions for the group. After the meeting, the participants formulate one or more goals for improvement to work on in the upcoming weeks.

**TABLE 5:** Final reflection method based on the analysis of this study.

Individual actions before each meeting
*Each participant*: Chooses a digital client record for reflection Assesses this digital client record using the quality criteria Assesses and gives feedback on the reporting in client records of two colleagues Reflects on the feedback given by two colleagues and formulates one or more questions to pose in the meeting

Steps within the meeting	
*Meeting 1* Participant 1 introduces digital file of client case	*Meeting 2* Participant 1 discusses personal goals and actions taken
2 Participant 1 discusses the received feedback 3 Participant 1 poses one or more questions 4 The group takes time to think about the questions and an overarching theme 5 Group discussion for defining an overarching theme 6 Group discussion on opportunities to address the overarching theme 7 Repeat steps 1–6 for each participant (Participants 2–6) 8 The group takes time to think about individual actions related to the discussed themes 9 Each participant formulates a personal goal for change and action plan10. The coach evaluates the meeting with the group (process and yields/gains) and summarises the refection method steps to be taken after the meeting	

Individual actions after the meeting	
*After meeting 1* Each participant formulates 1–3 learning goals and action plan and takes action	*After meeting 2* Each participant evaluates his/her progress

## 5 Discussion

In this study, a reflection method for implementation of a professional reporting guideline for community nurses and certified nursing assistants was developed, tested and evaluated. The close collaboration of end‐users with the research team resulted in a reflection method that matched the learning needs, preferences and context of the target group.

Three main themes and eight sub‐themes were identified referring to the impact on critical reflection and behaviour change. These themes were linked to seven key features of reflection, which could be used for the development of reflection methods for reflection on clinical guidelines. These key features clarified which elements were essential in developing a reflection method aimed at enhancing adherence to guidelines.

Participants in this study emphasised learning from viewing other teams' client reports, which can be qualified as more implicit (intuitive) learning. Learning from reflection, feedback and work samples was considered explicit (conscious) learning. Explicit learning is facilitated by instruction and deliberate practice; implicit learning is facilitated by experience and social interaction such as role modelling (Bolhuis 2016; Destrebecqz and Cleeremans 2001; Hodges and Franks 2002; Steenbergen et al. 2010). The final reflection method was designed to strengthen both explicit and implicit learning processes to match the differences among nurses.

An additional outcome of this study was that the reflection method stimulated more communication among participants outside the meetings, resulting in a positive effect on team spirit, particularly important because community nurses and certified nursing assistants typically work alone and rarely see each other. Seeing each other allowed them to discuss the quality of the client reports as well as the care given. Numerous studies have reported teamwork and relationships with colleagues as important factors determining the level of nurses' job satisfaction (Atefi et al. 2014; Majima et al. 2019; Van Bogaert et al. 2017; Wyatt and Harrison 2010). Teamwork and relationships with colleagues provide faster, safer and more efficient client care. Collaborative teamwork and two‐way communication between community nurses and certified nursing assistants promote patient safety (Campbell, Layne, and Scott 2021). The high relational quality between nurses and certified nursing assistants is an important component of teamwork and patient safety culture; joint responsibility, collaboration and prioritising and sharing patient needs are mentioned in a previous research as critical behaviours to achieve this (Campbell, Layne, and Scott 2021).

This study showed that this reflection method for the use of a professional reporting guideline is meaningful for several reasons. Worldwide, reflection in nursing practice is an accepted strategy for professional development. However, evidence of its clinical benefits remains limited. In other areas of expertise, such as physical therapy, there is more evidence that it is beneficial (Maas et al. 2015; van Dulmen et al. 2014). The individual nurse's reflective practice capability is essential in providing and improving nursing care by narrowing the gap between clinical practice and guidelines (Asselin and Fain 2013; Contreras et al. 2020; Gustafsson, Asp, and Fagerberg 2007; Rolfe 2014). Through the reflection method, they will learn to reflect individually and in a team setting on their actions, which can help narrow the gap between practice and guidelines.

### 5.1 Strengths and Limitations

The strength of this study is the research design. The design‐based approach allowed the reflection method to be developed with the end‐users, ensuring a match with their nursing practice (Hartswood et al. 2008; Peffer and Renken 2016). The reflection method is a result of a co‐creation process between researchers and end‐users. Obtaining participants' feedback on the research findings and co‐designing the reflection method add validity to the researcher's interpretations by ensuring that the participants' own beliefs and perspectives are represented and not constrained by the researchers' own agenda and knowledge (Reeves and McKenney 2015). The end‐users had different demographic information, such as age, education level, years of work experience and digital proficiency. Comparing this group's characteristics to that of community nurses in the Netherlands (Francke et al. 2017), it can be concluded that the participants represent this group very well, ensuring the link to their daily practice. It should be noted that no registered nurses or certified nursing assistants from different cultural backgrounds participated in this study. It is unclear whether this is due to them not being asked, not responding or being less represented within the participating home care organisations.

The second strength of this study is that a patient representative was affiliated with the design meetings to provide input for the reflection method from a patient perspective. The third strength of this study is that all data from design and test meetings were iteratively collected and analysed, resulting in relevant information and directions for improvement of the design identified and used in the next design phase. Moreover, triangulation was ensured by using different types of data to strengthen the credibility of the results (Tong, Sainsbury, and Craig 2007).

A limitation of this study is that most test meetings were conducted online due to the COVID‐19 pandemic, and only one session took place in person. During this session, participants expressed a preference for in‐person meetings, as they allow for more in‐depth discussions and relationship‐building. The study was not conducted as originally planned due to COVID‐19; however, despite the online meetings, it generated substantial data, leading to the identification of key features.

Another limitation of the study is the type of design, in that it produces results that are regarded as context‐specific and applicable only to the participating organisations. However, key features were established, which are expected to be transferable to other contexts.

### 5.2 Implication for Practice and Further Research

Reflection provides important benefits for better applying the guidelines in clinical practice (Contreras et al. 2020; Patel and Metersky 2022; Rolfe 2014; Wensing, Grol, and Grimshaw 2020). Therefore, it is recommended that this reflection method be implemented within home care organisations to reduce the gap between practice and reporting recommendations, in order to act more in accordance with this Reporting guideline. As a result, there will be less unwanted practice variation, and it will optimise the quality of client care (Graham et al. 2011; Spoon et al. 2020). For implementation of the reflection method within home care organisations, it might be useful to further identify barriers and facilitators and matching strategies.

In recent years, many countries, including the Netherlands, have experienced an increased workload for nurses due to staff shortages and changes in healthcare. This method was developed in direct collaboration with nurses and certified nursing assistants, tailored to their specific needs. During the development and in the evaluation, much attention was paid to embedding of the reflection method in daily work. Moreover, the reflection method has demonstrated its potential to improve both the quality of reporting and overall patient care. The reflection process allowed nurses and certified nursing assistants to develop practical strategies to streamline the delivery of care. By taking a more critical and organised approach to their daily practice, they can not only maintain efficiency but also improve it. Rather than increasing their workload, this reflection method helps more effective management of their existing responsibilities through critical reflection on their daily actions toward the guidelines.

This study developed a reflection method specific to a professional reporting guideline for nurses and certified nursing assistants. In further research, a transition must be made so that the reflection method can be used for guidelines in general. In addition, a follow‐up study could examine the effectiveness of the reflection method.

## 6 Conclusions

An evidence‐ and practice‐based reflection method for community nurses and certified nursing assistants for the implementation of a professional reporting guideline was developed. By involving community nurses and certified nursing assistants, the method closely matches their needs and preferences.

This reflection method consists of two 2 h meetings with up to six participants and a coach, in which they evaluate their own reporting and the reporting of colleagues based on self‐selected client reports. Key features are: help participants to focus on critical reflection, allocate time to formulate an overarching theme or problem that aligns with the learning needs of the entire group, include participants from different teams, varying in working experience, and educational levels, limit group size to six participants, allow for sufficient reflection time and stimulate the group to make the transfer of learning to their own clinical practice.

## Conflicts of Interest

The authors declare that they have no known competing financial interests or personal relationships that could have appeared to influence the work reported in this paper. The authors declare the following financial interests/personal relationships which may be considered as potential competing interests.

## Peer Review

The peer review history for this article is available at https://www.webofscience.com/api/gateway/wos/peer‐review/10.1111/jan.16747.

## Data Availability

Anonimised data will be available on request.
